# The Olfactory Receptor Family 2, Subfamily T, Member 6 (OR2T6) Is Involved in Breast Cancer Progression via Initiating Epithelial-Mesenchymal Transition and MAPK/ERK Pathway

**DOI:** 10.3389/fonc.2019.01210

**Published:** 2019-11-11

**Authors:** Ming Li, Xiao Wang, Ran-Ran Ma, Duan-Bo Shi, Ya-Wen Wang, Xiao-Mei Li, Jun-Yi He, Jun Wang, Peng Gao

**Affiliations:** ^1^Key Laboratory for Experimental Teratology of the Ministry of Education and Department of Pathology, School of Medicine, Shandong University, Jinan, China; ^2^Department of Pathology, Dezhou People's Hospital, Dezhou, China; ^3^Department of Pathology, Qilu Hospital, Shandong University, Jinan, China

**Keywords:** OR2T6, breast carcinoma, epithelial-mesenchymal transition, MAPK/ERK pathway, prognosis

## Abstract

Breast cancer is the most common female malignancy worldwide, however its molecular pathogenesis still needs in-depth investigation. Here we first revealed that the olfactory receptor family 2, subfamily T, member 6 (OR2T6) was significantly over-expressed in breast cancer tissues compared with normal breast tissues. OR2T6 expression was tightly correlated with higher TNM staging, positive lymph node metastasis, and associated with poorer patients' overall and disease-free survival. And OR2T6 enhanced the proliferation, invasion, and migration ability of breast cancer cell lines *in vitro* (MCF-7 and MDA-MD-231). Mechanically, it promoted the expression of mesenchymal markers (Vimentin, N-cadherin, and β-catenin) while inhibited E-cadherin expression, suggesting that OR2T6 played a key role in the regulation of epithelial-mesenchymal transition (EMT) process. Moreover, the human gene expression microarray clarified that MAPK/ERK pathway could be initiated by OR2T6 at mRNA level, which was further confirmed at protein level by western blot analysis. Thus, we concluded that OR2T6, as a novel oncogene, contributed to the progression of breast carcinoma by the initiation of EMT and MAPK/ERK pathway.

## Introduction

Breast cancer is the most common cancer and the fifth leading cause of cancer death among women worldwide. More than 1,000,000 new cases are diagnosed annually and ~373,000 result in death each year (1, 2). Although breast cancer mortality has declined in the last few decades as a consequence of advanced screening programs and adjuvant therapies, some patients are still at high risk of relapse and have poor prognoses (2–4). The detailed molecular mechanism involved in the carcinogenesis of breast cancer remains unclear (1).

Recently, we used a gene microarray method to analyze the differential expression between cancers with and without metastasis (GEO accession number: GSE72307) (5). We identified that the olfactory receptor family 2, subfamily T, member 6 (OR2T6) was significantly higher in cancers with metastasis than in those without metastasis. This result indicated that OR2T6 might play a role in the progression of human solid cancers. However, the function of OR2T6 in human malignancies has not been reported.

The olfactory receptor (OR) family is a key member of the G-protein-coupled receptor (GPCR) family in humans (6, 7). The majority of ORs are specifically expressed in the sensory neurons of the olfactory epithelium, but some ORs are also expressed in non-olfactory tissues (8–11). Recently, OR51E2 [prostate-specific G-protein coupled receptor (PSGR)] was found to be overexpressed in prostate cancer tissues (12), and it accelerates prostate cancer development together with the loss of PTEN in a mouse model (13). Olfactory receptor family 2 subfamily AT member 4 (OR2AT4) was reported to regulate cell proliferation, apoptosis, and differentiation in human myelogenous leukemia (14). Olfactory receptor family 7 subfamily C member 1 (OR7C1) was also found to be a novel marker for colon-cancer-initiating cells (CICs) (15). Thus, ORs might play a vital role in human malignancies. However, in spite of these observations, the function of the OR family and the underlying molecular mechanisms are still largely unknown.

In the present study, to verify the possible role of OR2T6 in breast cancer, we tested the mRNA and protein levels of OR2T6 in breast cancer tissues *in vivo*; elucidated its functional role in breast cancer cell proliferation, invasion, and migration *in vitro*; and clarified its possible involvement in the regulation of the epithelial-mesenchymal transition (EMT) and the mitogen-activated protein kinase/extracellular regulated protein kinases (MAPK/ERK) pathway.

## Materials and Methods

### Ethics Statement

This study was approved by the ethics committee of the Medical School of Shandong University (approval code: 2012028). Written informed consent was obtained from all patients before the use of the materials. All procedures performed in studies involving human participants were in accordance with the ethical standards of the institutional and/or national research committee and with the 1964 Helsinki declaration and its later amendments or comparable ethical standards.

### Patient Population

We collected 102 samples of breast cancer tissue from patients who underwent modified radical mastectomy because of breast cancer and 60 cases of normal breast tissue from patients who underwent local resection because of fibrocystic disease of the breast at Qilu Hospital of Shandong University between July 2005 and July 2012. None of the patients had received radiotherapy or chemotherapy prior to surgery. Hematoxylin and eosin (HE)-stained sections and paraffin-embedded tissue sections were obtained from the Department of Pathology, and patient information was obtained from the patient medical record room at Qilu Hospital. All of the HE sections were reviewed by two experienced pathologists, and the diagnoses were classed according to the World Health Organization Classification of Tumors.

### Follow-Up

Patients were followed up every 3 months until death or July 2017. Survival time, disease-free interval, and development of metastases were recorded as survival data. The interval between the dates of surgery and death was used as the overall survival (OS) time. The interval between the date of the operation and recurrence or death was recorded as the disease-free survival (DFS) time. Patients still alive at the last follow-up were classified as censored observations.

### Exploring Cancer Genomics Data Using Cbioportal

The cBioPortal for cancer genomics (http://www.cbioportal.org/) provides analysis and download of large cancer genomics datasets. In this study, we used cBioPortal to explore the genetic variation of OR2T6 in breast cancer.

### Cell Lines and Culture

The human breast cancer cell lines MCF-7 and MDA-MB-231 were purchased from the American Type Culture Collection (Manassas, VA, USA) (last authenticated in April 2018 using STR profile analysis). Cells were cultured per routine in Dulbecco's modified Eagle's medium (MCF-7) or Leibovitz's L15 medium (MDA-MB-231) with 10% fetal bovine serum (FBS, Gibco BRL, Grand Island, NY, USA) and 1% penicillin-streptomycin at 37°C in a humidified atmosphere with 5% CO_2_.

### RNA Extraction and Real-Time PCR

Total RNA was extracted using Trizol (Ambion, cat. 15596-026) and synthesized into cDNA with the RevertAid First Strand cDNA Synthesis kit (Thermo Fisher Scientific, cat. K1621). The detailed primer sequences were listed in Supplementary Table S1. GAPDH was used as the internal control. Real-time PCR was performed on the ABI Prism 7000 Sequence Detection System with SYBR Premix Ex Taq (Takara, RR420A).

### Plasmid Construction, Small-Interfering RNA (siRNA), and Plasmid Transfection

For plasmid construction, the whole OR2T6 sequence was synthesized and inserted into the pcDNA3.1 vector at the Nhel/Xhol restriction enzyme site for the construction of the pcDNA3.1-OR2T6 plasmid (Boshang Biological Technology, Shanghai, China). The pcDNA3.1 vector was used as the control. The siRNA sequences were as follows: OR2T6, 5′-GCUCUUCACUCACAAUAAAdTdT-3′ and NControl_05815 (siN05815122147) (Ribobio, Guangzhou, China). MCF-7 and MDA-MB-231 cells were seeded in 6-well culture plates at 2 × 10^5^ cells per well and cultured in medium without antibiotics for 24 h before transfection. Lipofectamine 2000 (Invitrogen, Carlsbad, CA, USA) was used to transfect the siRNAs or plasmids into cells.

### Western Blot Analysis

Total protein was extracted from cells using CelLytic^TM^ MT Cell Lysis Reagent (Sigma, cat. C3228) and qualified using the BCA reagent kit (Beyotime, cat. P0011). Protein was loaded onto 10% gels for immunoblotting with antibodies against OR2T6, E-cadherin, N-cadherin, β-catenin, vimentin, H-RAS, c-Myc, ERK, c-fos, or β-actin separately. β-actin was used as the loading control. Detailed information about the antibodies was listed in Table 1. The protein bands were visualized using an enhanced chemiluminescence kit (Beyotime, cat. P0018S).

**Table 1:** Primary antibodies used in this study.

**Antibody**	**Application**	**Dilution**	**Supplier**	**Source**	**Monoclonal or polyclonal**	**Catalog number**
OR2T6	WB, IHC-P	WB:1:1000, IHC-P:1:150	Santa Cruz	Goat	Polyclonal	sc-104547
H-RAS	WB	1:300	Sangon Biotech	Rabbit	Polyclonal	D260079
c-Myc	WB	1:1000	Sangon Biotech	Rabbit	Polyclonal	D110006
ERK	WB	1:2000	CST	Rabbit	Monoclonal	4370S
c-fos	WB	1:500	Sangon Biotech	Rabbit	Polyclonal	D120415
E-cadherin	WB	1:1000	CST	Rabbit	Monoclonal	mAb 3195
N-cadherin	WB	1:1000	CST	Rabbit	Monoclonal	mAb13116
β-catenin	WB	1:1000	CST	Rabbit	Monoclonal	mAb 8480
Vimentin	WB	1:1000	CST	Rabbit	Monoclonal	mAb 5741

### Proliferation Assay

Cell proliferation was measured using Cell-Light™ EdU DNA Cell Proliferation (EdU) assays (Ribobio, cat. C10310). Briefly, cells were seeded onto 96-well plates at a density of 5 × 10^3^ cells per well at 24 h after transfection. After EdU labeling, cells were treated with 100 μL of 1× Apollo reaction cocktail, stained with 100 μL of Hoechst 33342 (5 μg/mL), and observed under a fluorescence microscope (Olympus, Japan). The proliferation rate was determined by calculation of the percentage of EdU-positive cells.

### Cell Apoptosis Assay

Cells were collected at 72 h after transfection and stained with the Annexin V-FITC/PI Apoptosis Detection Kit (BestBio, cat. BB4101), according to the manufacturer's instructions. Cells with negative PI and positive FITC staining were defined as early apoptotic cells, while those with positive PI and FITC staining were defined as late apoptotic cells. The total apoptotic rate was calculated as the early apoptotic rate plus the late apoptotic rate.

### Cell Migration and Invasion Assays

Migration assays were conducted using Transwell inserts (8.0 μm, 24-well format; Corning, NY, USA). For the invasion assay, the inserts were pre-coated with a Matrigel matrix (BD Science, Sparks, MD, USA). Cell migration and invasion assays were performed as described previously (16).

### Human Gene Expression Microarray

MCF-7 cells were transfected with OR2T6 siRNA or negative control and then subjected to human gene expression microarray (KangCheng Inc., Shanghai, China) assay. Genes with a fold-change above two after transfection were defined as differentially expressed genes (DEGs). Bioinformatic analyses, namely, DEGs gene ontology (GO) enrichment analysis, together with Kyoto Encyclopedia of Genes and Genomes (KEGG) pathway enrichment analysis, were performed using the Database for Annotation, Visualization, and Integrated Discovery (DAVID) online tool (http://david.abcc.ncifcrf.gov/).

### Immunohistochemistry Staining (IHC)

An immunohistochemical method was used to detect the protein expression in 162 tissue samples. Briefly, slides were baked at 60°C, deparaffinized, and rehydrated. After antigenic retrieval, the sections were treated with 3% hydrogen peroxide and incubated with normal serum. Then, slides were further incubated with antibodies for OR2T6 overnight at 4°C. On the second day, the slides were treated with reagent 1 containing the secondary antibody and reagent 2 containing streptavidin-horseradish peroxidase complex (PV-9000 kit, Zhongshan Biotechnology Company, Beijing, China) for 30 min at room temperature separately. After diaminobenzidine (DAB) staining, the sections were counterstained with hematoxylin. PBS buffer was used as a negative control to replace OR2T6 antibody.

The staining results were independently judged by two pathologists blinded to the patients' information. Staining index (SI) was used to determine the results by combining the staining intensity and the proportion of positive cells. In brief, staining intensity was graded as follows: 0 (no staining), 1 (light yellow), 2 (yellow brown), and 3 (strong brown). The positive proportion of tumor cells was evaluated as follows: 0 (positive cells <5%); 1 (positive cells: 5–25%); 2 (positive cells: 26–50%); 3 (positive cells: 51–75%); and 4 (positive cells >75%). Then, the final SI scores were graded as 0, (-); ≤ 5, (+); 6–8, (++); and ≥ 9, (+++). Tumor samples scored (-) to (+) were considered negative, and others were considered positive.

### Statistical Analysis

SPSS 17.0 software (SPSS Inc., Chicago, IL, USA) was used for statistical analysis. The chi-square test was used to evaluate the difference in OR2T6 expression between normal breast and breast cancer tissues. Student's *t*-test were implemented to compare the differences between the two groups. The chi-square test and Fisher's exact test were used to assess the relationship between OR2T6 expression and the clinicopathologic characteristics. The Kaplan-Meier method and log-rank test were used to analyze the patients' survival. Univariate and multivariate analyses involving Cox proportional hazard regression models were further performed to evaluate whether OR2T6 could be an independent indicator of prognosis. *P* < 0.05 was considered statistically significant.

## Results

### OR2T6 Is Overexpressed in Breast Cancer Specimens

**Figure 1:**
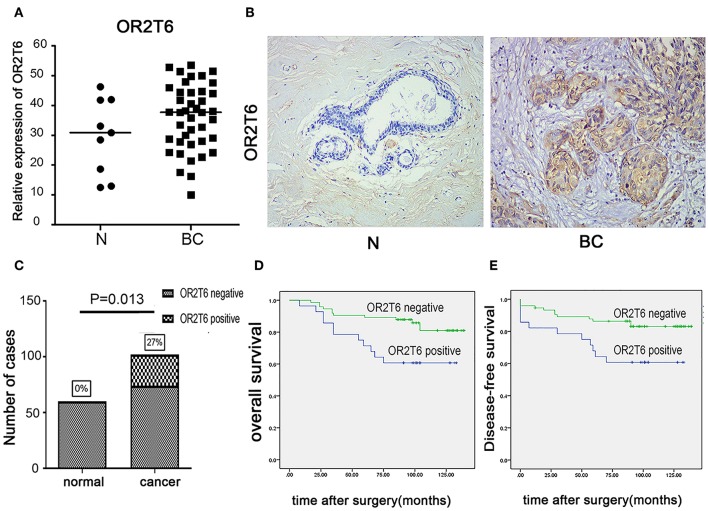
OR2T6 expression in breast cancer tissues and its association with patients' outcome. **(A)** The mRNA levels of OR2T6 in normal breast tissues (***n*** = 9 samples) and breast cancer tissues (***n*** = 41 samples) were assessed by the method of real-time quantitative PCR. Chi-square test was used to analyze the difference between the two groups. **(B)** Representative immunohistochemistry staining of OR2T6 protein in normal breast tissues (***n*** = 60 samples) and breast cancer tissues (***n*** = 102 samples) (H&E magnification, 40×). OR2T6 protein located in the cytoplasm. “BC” stands for breast cancer; “N” stands for normal breast epithelium. **(C)** Chi-square test revealed that OR2T6 expression was significantly higher in breast cancer tissues than in normal tissues. **(D,E)** Survival was analyzed by the Kaplan–Meier method and compared by log-rank tests. Kaplan-Meier curves for survival of 102 patients with breast cancer by OR2T6 positivity and negativity. Culminative overall survival **(D)** and disease-free survival **(E)**.

We first examined the mRNA levels of OR2T6 in normal breast tissues (*n* = 9 samples) and breast cancer tissues (*n* = 41 samples) by real-time quantitative PCR. Results showed that the transcriptional levels of OR2T6 were significantly higher in breast cancer tissues than in normal ones (Figure 1A; *p* < 0.05). Furthermore, we found no signal in the 60 normal breast tissue samples by the IHC method, which indicated low expression of OR2T6 in normal breast duct epithelium (Figure 1B). In contrast, cytoplasmic OR2T6 was detected in 27.45% (28/102) of breast cancer tissues (Figure 1B). The protein level of OR2T6 was significantly higher in breast cancer tissues than in normal tissues (*p* = 0.013) (Figure 1C).

### The Association of OR2T6 Expression With Clinicopathological Parameters and Its Role in Determining the Prognosis of Breast Cancer Patients

We next investigated the correlation of OR2T6 expression with the clinicopathological characteristics of breast cancer patients. Statistical analysis showed that the positive expression of OR2T6 was tightly associated with lymph node metastasis (*p* = 0.002) and higher TNM (Tumor/Node/Metastasis) staging (*p* = 0.033), but not with patients' age, tumor size, or histological grade (Table 2). Furthermore, Kaplan-Meier survival analysis and log-rank test revealed that patients with OR2T6 expression had poorer overall survival (97 months vs. 124 months, *p* = 0.009) and disease-free survival (92 months vs. 121 months, *p* = 0.013) than those without OR2T6 expression (Figures 1D,E). In an univariate analysis, OR2T6 was a significant predictor for higher hazard ratio (HR), together with tumor staging and lymph node metastasis (all of the *p*-values <0.05; Table 3); however, it was not an independent prognosis factor in multivariate analysis (Table 3).

**Table 2:** Association of clinicopathological parameters with OR2T6 protein expression.

	**Number of cases**	**OR2T6**		***P* value**
		**Positive**	**Negative**	
**Age**				0.821
<50	40	10	30	
≥50	62	18	44	
**LN metastasis**				**0.002**
No	48	6	42	
Yes	54	22	32	
**Tumor size**				0.503
≤2 cm	43	10	33	
>2 cm	59	18	41	
**TNM staging**				**0.033**
I	24	4	20	
II	55	13	42	
III	14	5	9	
IV	9	6	3	
**Histological grade**				0.095
I	7	0	7	
II	76	20	56	
III	19	8	11	

*LN, lymph node; P < 0.05 was considered statistically significant (indicated in bold)*.

**Table 3:** Univariate and multivariate Cox proportional hazards regression analysis of survival in 102 patients with breast cancer.

	**Univariate analysis**			**Multivariate analysis**		
	**HR**	**CI (95%)**	***P*-value**	**HR**	**CI (95%)**	***P*-value**
**Overall survival**						
OR2T6 expression	2.831	1.246–6.431	**0.013**	1.362	0.490–3.786	0.554
Histological Grade	2.006	0.899–4.475	0.089	2.716	1.081–6.824	**0.034**
LN metastasis	4.759	1.615–14.027	**0.005**	1.781	0.513–6.182	0.363
TNM staging	5.035	2.978–8.513	**0.000**	5.728	3.254–10.084	**0.000**
Tumor size	2.348	0.925–5.958	0.072	1.209	0.394–3.708	0.741
Age	2.079	0.910–4.750	0.082	2.458	1.050–5.751	**0.038**
**Disease-free survival**						
OR2T6 expression	2.691	1.186–6.102	**0.018**	1.275	0.480–3.388	0.626
Histological grade	1.852	0.841–4.078	0.126	2.039	0.832–4.994	0.119
LN metastasis	5.109	1.736–15.038	**0.003**	4.228	1.403–12.745	**0.010**
TNM staging	5.322	3.115–9.091	**0.000**	2.265	0.820–6.256	0.115
Tumor size	2.395	0.944–6.078	0.066	1.213	0.386–3.809	0.741
Age	2.140	0.938–4.883	0.071	1.566	0.655–3.742	0.313

*LN, lymph node; HR, hazard ratio; 95% CI, 95% confidence interval. P < 0.05 was considered statistically significant (marked in bold)*.

### Genetic Alterations of OR2T6 Gene in Breast Cancer

We used cBioPortal to study the genetic changes of OR2T6 gene in 10 breast cancer studies. In 8,023 cases of breast cancer, the genetic change rate of OR2T6 ranged from 0 to 22.23%, with an average of 9.21%. Of 743 cases of genetically altered breast cancer, 734 cases had OR2T6 amplification, 5 cases had mutation, and 4 cases had deep deletion (Supplementary Figure S1). In Pereira B's study (17), the proportion of such genetic changes was as high as 22.23% (483/2173) (Supplementary Figure S1, left column).

### OR2T6 Promotes the Proliferation and Inhibits the Apoptosis of Breast Cancer Cells

**Figure 2:**
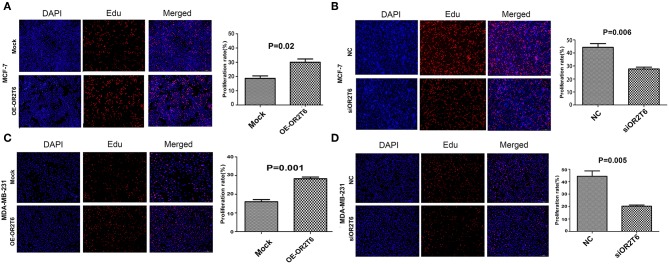
OR2T6 promotes the proliferation of breast cancer cell lines MCF-7 and MDA-MB-231. The percentage of EdU positive cells was recorded as the proliferation rate. Student's *t*-test showed that the proliferation rate was increased significantly when OR2T6 was over-expressed in breast cancer cells (**A,C**), and reduced dramatically when OR2T6 was knocked down **(B,D)**. Results were representative of triplicate experiments. “Mock” and “OE-OR2T6” stand for the cells transfected with pcDNA3.1 vector and pcDNA3.1-OR2T6 plasmid, respectively. “NC” and “siOR2T6” stand for the cells transfected with negative control siRNA and specific siRNA targeting the OR2T6 gene, respectively.

To explore the role of OR2T6 in the proliferation and apoptosis of breast cancer cells, we performed EdU staining and flow cytometry assays in MCF-7 and MDA-MB-231 cells. The results showed that the overexpression of OR2T6 in MCF-7 cells significantly increased the percentage of EdU-positive cells, which are the proliferating cells (Figure 2A) (*p* = 0.02). Consistent with this, the interference of OR2T6 notably decreased the percentage of proliferating cancer cells (Figure 2B) (*p* = 0.006). Similar results were obtained in MDA-MB-231 cells (Figures 2C,D) (*p* = 0.001, OE-OR2T6 vs. mock group; *p* = 0.005, siOR2T6 vs. NC group). Thus, OR2T6 enhanced the proliferation of breast cancer cells.

**Figure 3:**
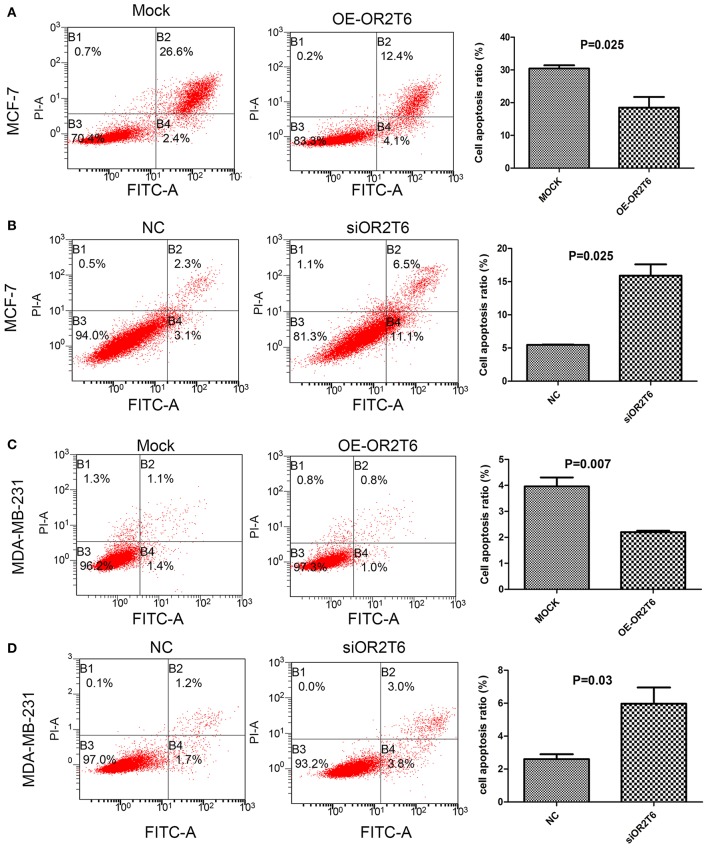
OR2T6 inhibited apoptosis of breast cancer cell lines. Cells were collected at 72 h after transfection, stained with Annexin V-FITC and PI and then analyzed by flow cytometry. Apoptosis of MCF-7 and MDA-MB-231 cells was inhibited by overexpression of OR2T6 **(A,C)** and enhanced by interference with OR2T6 expression **(B,D)**. Student's *t*-test was implemented to compare the difference between the two groups. Results were representative of triplicate experiments.

For the apoptosis assay, when we increased OR2T6 expression in MCF-7 cells, the percentage of apoptotic cells was reduced from 29.0 to 16.5% (Figure 3A) (*p* = 0.025), and when we knocked down OR2T6 expression, the proportion of apoptotic cells was elevated from 5.4 to 17.6% (Figure 3B) (p = 0.025). Similarly, changes in OR2T6 expression in MDA-MB-231 cells also affected the cell apoptosis rate significantly (Figures 3C,D) (*p* = 0.007, OE-OR2T6 vs. mock group; *p* = 0.03, siOR2T6 vs. NC group). The data indicated that OR2T6 inhibited apoptosis, thereby facilitating the transformation of breast cancer cells.

### OR2T6 Enhances the Migration and Invasion of Breast Cancer Cells via Regulation of EMT Progression

**Figure 4:**
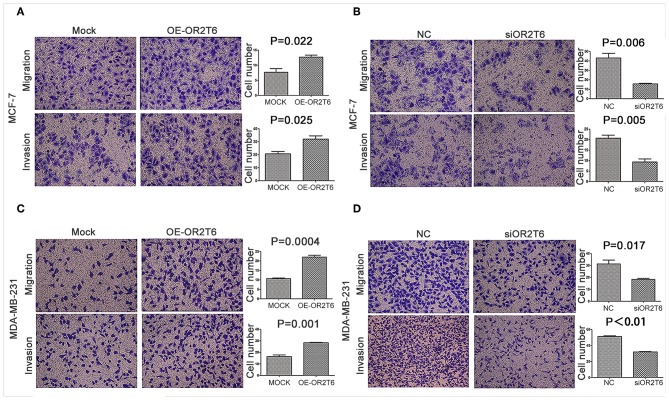
OR2T6 enhances cell migration and invasion of breast cancer cell lines. Cells (1 × 10^5^) were seeded in 12-well plates the day before transfection. After 24 h of transfection, the cells (1 × 10^4^) were resuspended in serum-free medium and seeded to the upper chamber of a Boyden chamber Transwell. Culture medium containing 10% fetal bovine serum was added to the lower chamber. Then, 36 h later, any cells that had migrated were fixed, stained, and counted. For the invasion assay, an experiment was done using membranes coated with a Matrigel matrix. The migration and invasion of MCF-7 and MDA-MB-231 cells were increased by overexpression of OR2T6 **(A,C)** and decreased by interference with OR2T6 expression **(B,D)**. Student's *t*-test was used to compare the difference between the two groups. Results were representative of triplicate experiments.

To assess the effects of OR2T6 on the invasion and migration capability of breast cancer, we first transfected breast cancer cells with pcDNA3.1-OR2T6 plasmid. The results showed that the overexpression of OR2T6 significantly enhanced the migration and invasion of MCF-7 cells (Figure 4A) (*p* = 0.022 and 0.025, respectively) and MDA-MB-231 cells (Figure 4C) (*p* = 0.0004 and 0.001, respectively). Furthermore, when we transfected breast cancer cells with siRNA specific to OR2T6, migration, and invasion were dramatically reduced in MCF-7 cells (Figure 4B) (*p* = 0.006 and 0.005, respectively) and MDA-MB-231 cells (Figure 4D) (*p* = 0.017 and *p* < 0.01, respectively). These data demonstrated that OR2T6 strengthened the migration and invasion of breast cancer cells.

**Figure 5:**
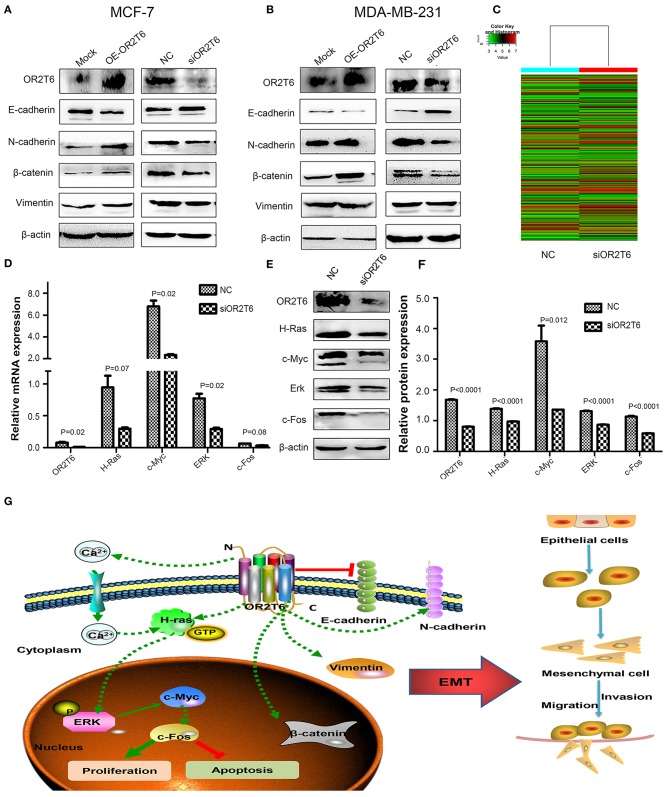
OR2T6 plays a role in the regulation of the EMT process and the MAPK/ERK pathway in breast cancer cells. **(A,B)** OR2T6 promotes the initiation of the EMT in breast cancer cells. The overexpression of OR2T6 reduced the expression of E-cadherin while increased those of N-cadherin, β-catenin, and vimentin in MCF-7 **(A)** and MDA-MB-231 cells **(B)**. When OR2T6 was knocked down, the opposite results were observed **(A,B)**. β-actin was used as the loading control. **(C)** Hierarchical clustering of MCF-7 cells transfected with negative control siRNA (NC) and specific siRNA targeting OR2T6 (siOR2T6) on the basis of differentially expressed mRNAs. Each row represents the expression level of an individual mRNA. Pseudo-colors indicate expression levels from low to high (green to red). (D) Real-time PCR data showed that the relative mRNA levels of genes in the MAPK/ERK pathway were reduced after silencing of OR2T6 expression in MCF-7 cells. **(E,F)** Western blot analysis revealed that the relative protein levels of genes in the MAPK/ERK pathway were decreased after transfection with siOR2T6. Student's *t*-test was used to compare the difference between the two groups. Each experiment was performed in triplicate. **(G)** Schematic model of the potential function mechanism of OR2T6 in breast cancer. The over-expression of OR2T6 enhances migration and invasion by initiating the EMT, and promotes proliferation and inhibit apoptosis via activation of the MAPK/ERK pathway.

To clarify the underlying mechanism responsible for the effects of OR2T6 on cell migration and invasion, we investigated the role of OR2T6 in the EMT process. When we transfected the pcDNA3.1-OR2T6 plasmid into MCF-7 cells, the protein expression of OR2T6 increased significantly, indicating the specificity of the vector (Figure 5A). Along with the increase of OR2T6, the expression of the epithelial marker E-cadherin decreased dramatically, while the mesenchymal markers, such as vimentin, N-cadherin, and β-catenin increased significantly (Figure 5A). In contrast, when we transfected OR2T6 siRNA into MCF-7 cells, the expression of the epithelial marker was enhanced, while that of the mesenchymal markers was significantly inhibited (Figure 5A). Similar results were obtained after the alteration of OR2T6 expression in MDA-MB-231 cells (Figure 5B). Thus, OR2T6 plays an essential role in the promotion of the EMT process, and it might promote the migration and invasion of breast cancer cells via the initiation of EMT progression.

### OR2T6 Plays a Role in the Regulation of the MAPK/ERK Pathway

We next investigated the molecular signaling pathways via which OR2T6 functions in breast cancer. To achieve this, we transfected MCF-7 cells with OR2T6 siRNA and subjected them to human gene expression microarray assays. The mRNA levels of 28,186 genes were compared between the cells transfected with siOR2T6 and negative control. Bioinformatic analysis revealed that 13,452 genes were up-regulated, while 14,734 genes were down-regulated in the siOR2T6 group compared with the control group (data not shown). Furthermore, 385 differentially expressed genes (DEGs) were identified, among which 213 genes were up-regulated, and 172 genes were down-regulated after the interference with OR2T6 gene expression (Figure 5C). The complete microarray data are listed in the GEO database (accession number GSE108006).

To obtain a more comprehensive knowledge of the DEGs, GO, and KEGG pathway enrichment analysis were performed. GO analysis showed that the DEGs were mainly enriched in regulation of biological process, single-organism process, cellular response to chemical stimulus, and biological adhesion. KEGG analysis suggested that the DEGs were enriched in cAMP, calcium signaling pathway and pathways in cancer including Rap1, PI3K-AKT, and Ras signaling pathways. To verify the microarray data, real-time quantitative PCR was conducted in MCF-7 cells to validate the related molecules in Rap1 signaling pathway (Rap1A, Rap1B, MLLT4, CTNND1)and PI3K-AKT pathway (PIK3R5, AKT2, AKT3, BAD, and Bcl-2). However, results showed that the expression of these genes were not changed obviously (Supplementary Figure S2). We next detected the molecules involved in Ras signaling pathway. As expected, the mRNA levels of H-Ras, c-Myc, ERK, and c-fos genes, which belong to the MAPK/ERK pathway, were decreased after interference with OR2T6 expression (Figure 5D). Western blot analysis further proved that when we knocked down OR2T6 gene expression, the protein signals of the above genes were reduced significantly (Figures 5E,F). These results indicated that OR2T6 might function in the up-regulation of the MAPK/ERK pathway.

## Discussion

Although different ORs have different copy numbers and different functional alleles, highly related ORs often cluster at the same locus, indicating their specific functions in humans (18–20). It has been observed that PSGR facilitates cancer cell proliferation, invasion, and migration (21), while OR2AT4 and OR51B4 hamper cancer cell behaviors (14, 22). Therefore, ORs might have a direct role in carcinogenesis, and different ORs might play different roles in human malignancies.

It is well-known that the truncating mutation 1100delC of checkpoint kinase 2 *(*CHEK2) increases the risk of breast cancer about 2-fold. In 2011, Taru A. Muranen reported that 34 OR genes were overexpressed in breast tumors with the CHEK2 1100delC mutation. This implied that ORs might have a role in breast cancer (23). However, the function of ORs in breast cancer has not been clarified. Here, we demonstrated that OR2T6 enhanced the proliferation, invasion, and migration of breast cancer cells and inhibited apoptosis of these cells. OR2T6 expression in breast cancer tissues was significantly higher than that in normal breast tissues, and its expression was tightly associated with tumor staging and lymph node metastasis. Moreover, cBioPortal analysis confirmed the genetic alterations of OR2T6 gene in large-scale of breast cancer. Thus, we speculated that OR2T6 was a novel cancer-related OR, and it might play a role in the maintenance of the malignant phenotype of breast cancer.

The epithelial-mesenchymal transition (EMT) is a process by which epithelial cells lose their cell polarity and cell-cell adhesions and gain migratory and invasive properties (24). EMT is essential in the initiation of metastasis for cancer progression including breast cancer (25, 26). Weber et al. reported that incubation of colorectal cancer cells with Troenan, an effective ligand of OR51B4, leads to obvious morphological changes in cell shape (22). Zhang et al. also showed that OR2A4 is associated with cell migration by regulating the cytoskeleton of HeLa cells (27). This suggests that ORs could favor cell invasion and migration by regulating the actin cytoskeleton in cancer cell lines. However, the underlying mechanism remains unclear. In our research, we showed that alteration of OR2T6 expression significantly changed the protein levels of the epithelial and mesenchymal markers in breast cancer cells. This result provided strong evidence that OR2T6 had a vital role in the initiation of the EMT process, by which it might exert the function of facilitating cell invasion and migration.

The MAPK/ERK pathway is one of the most important for tumor initiation. Once the pathway is activated, tumor cell proliferation is greatly enhanced, and apoptosis is inhibited (28). Based on high-throughput information analysis, we preliminarily demonstrated a possible regulatory role of OR2T6 in the MAPK/ERK pathway. Interference with OR2T6 expression dramatically triggered the cascade of the MAPK/ERK pathway, and the expression of H-Ras, c-Myc, ERK, and c-fos was decreased significantly. It has been reported that the olfactory receptor can bind and activate G protein, which allows calcium ions to enter the cell and stimulate RAS gene expression (29–31). In our study, OR2T6 might have activated H-Ras gene expression through the regulation of calcium ions, and H-Ras thereby would have converted inactive guanosine diphosphate (GDP)-bound conformations to active guanosine triphosphate (GTP)-bound conformations, stimulating downstream ERK, c-Myc, and c-fos proteins (32, 33). Thus, OR2T6 might promote proliferation and inhibit the apoptosis of breast cancer cells via the initiation of the MAPK/ERK pathway.

Recent reports have shown three patterns of subcellular protein localization of ORs. OR51E1 protein was observed in the membrane and cytoplasm of epithelial cells (33). The majority of OR51E1 protein located in the cytoplasm, while others in the perinuclear compartment of tumor cells (34, 35). The diverse locations of one olfactory receptor suggest its diverse effects in different kinds of tissues. Moreover, variable ORs might have different subcellular localization and functions. In our present study, 28 of 102 cases of breast cancer showed cytoplasmic OR2T6 localization, while one case exhibited membranous localization. It indicates the variable roles of OR2T6 in breast cancer. However, the number of cases enrolled in this study was limited, and the precise subcellular position needs to be clarified in large cohorts of patients. The underlying mechanism also needs in-depth investigation.

OR2T6 belongs to GPCRs, the largest family of cell-surface receptors. GPCRs have been proved to participate in the development and progression of many tumors including breast cancer (36). Consistently, GPCR-based drugs have shown promising efficacy in halting the tumor growth. At present, 21 anti-cancer drugs targeting 14 different GPCRs have been approved. In clinical trials, 23 GPCR-targeted drugs are being developed for cancer treatment (36). However, the majority of human GPCRs were not targets for drugs because of our limited knowledge of their signaling mechanisms (37). In the present study, our findings in OR2T6 may help to broaden the molecular mechanism of GPCRs-induced breast cancer and to develop new therapeutic strategies for breast cancer patients in the future.

In the present study, we identified that OR2T6, as a new oncogene, might be involved in the initiation and progression of breast cancer. Its over-expression was associated with higher TNM staging and lymph node metastasis. Up-regulation of OR2T6 expression in breast cancer may enhance migration and invasion by initiating the EMT, and promote proliferation and inhibit apoptosis via activation of the MAPK/ERK pathway (Figure 5G). Considering the multifaceted functions of OR2T6, future approaches targeting OR2T6 might have promising potential in cancer treatment.

## Data Availability Statement

The datasets generated for this study can be found in the GEO database (accession number GSE108006).

## Ethics Statement

The studies involving human participants were reviewed and approved by the ethics committee of the Medical School of Shandong University (approval code: 2012028). The patients/participants provided their written informed consent to participate in this study.

## Author Contributions

PG conceived the experiments and wrote the paper. ML and XW carried out the *in vitro* experiments. R-RM and D-BS analyzed the data. Y-WW and X-ML carried out the *in vivo* experiments. J-YH and JW collected the specimens.

### Conflict of Interest

The authors declare that the research was conducted in the absence of any commercial or financial relationships that could be construed as a potential conflict of interest.
