# Supplementary material for: The Olfactory Receptor Family 2, Subfamily T, Member 6 (OR2T6) Is Involved in Breast Cancer Progression via Initiating Epithelial-Mesenchymal Transition and MAPK/ERK Pathway
Source: Front Oncol. 2019 Nov 11;9:1210. doi: 10.3389/fonc.2019.01210 (PMC6859866; doi:10.3389/fonc.2019.01210)
Supplement: Supplementary file 1 [file Data_Sheet_1.PDF]

Table S1. The primer sequences used in the real-time PCR.

Gene names	The primer sequences
OR2T6	Forward: 5'-ATGATCCTGGCCAGCTCTTG-3'<br>Reverse: 5'-TTGCAACGCAGCACACATAC-3'

Figure S1.

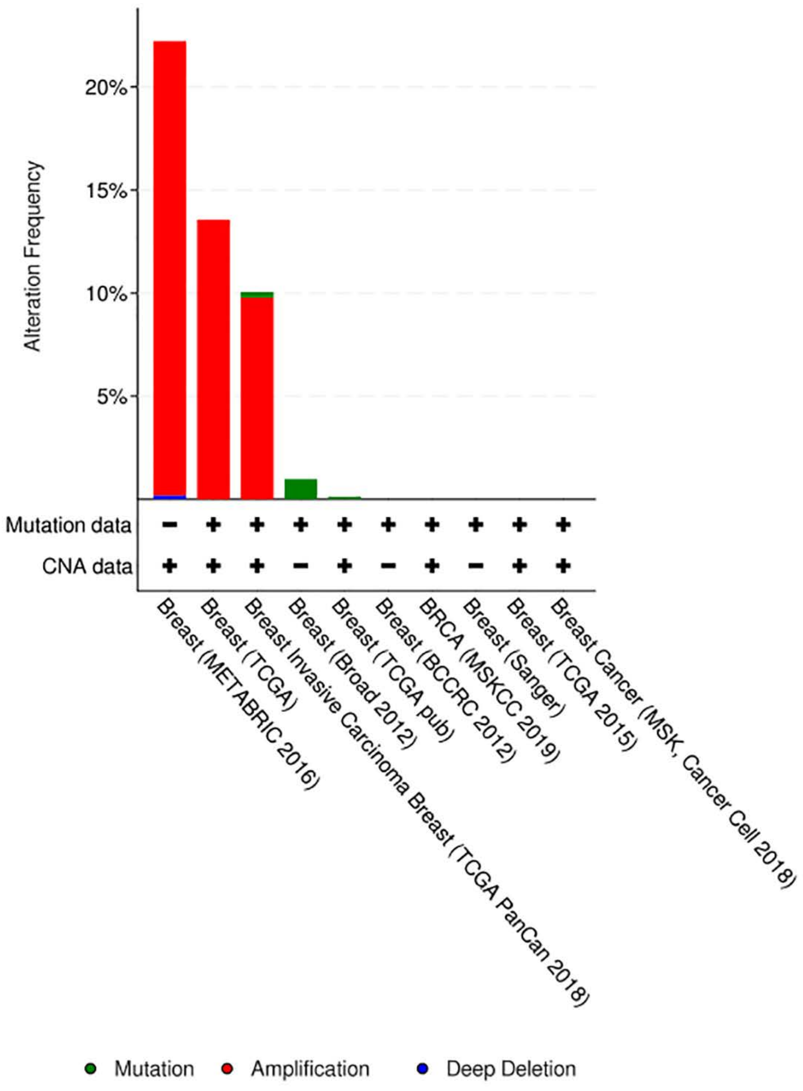

Figure S1. The genetic alteration of OR2T6 in breast cancer.

The cBioPortal analysis revealed that the genetic changes of OR2T6 ranged from 0% to 22.23%, with an average of 9.21% in 10 breast cancer studies. Of 743 cases of genetically altered breast cancer, 734 cases had OR2T6 amplification, 5 cases had mutation, and 4 cases had deep deletion. In Pereira B's study, the proportion of such genetic changes was as high as 22.23% (483/2173) (Left column).

Figure S2.

(A)

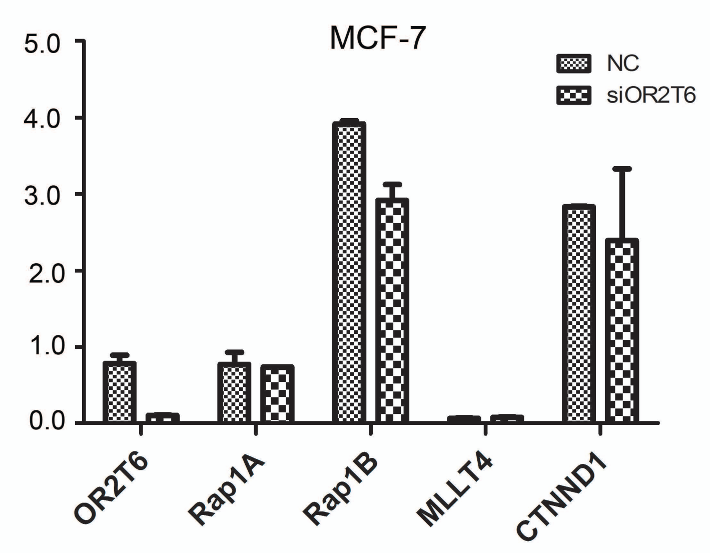

(B)

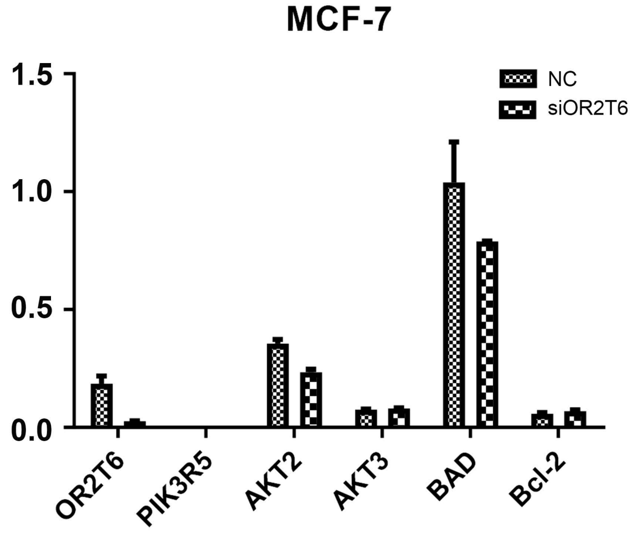

Figure S2. Silencing the expression of OR2T6 in MCF-7 cells had no significant effect on the expression of related molecules in RAP1 and PI3K-AKT signaling pathways. MCF-7 cells were transfected with the siRNA targeting OR2T6. Real-time quantitative PCR was conducted to validate the mRNA levels of the related molecules in Rap1 signaling pathway (Rap1A, Rap1B, MLLT4, CTNND1）and PI3K-AKT pathway (PIK3R5, AKT2, AKT3, BAD and Bcl-2). Results showed that the expression of these genes were not changed significantly after silencing OR2T6. Student's *t*-test was used to compare the difference between the two groups. Results were representative of triplicate experiments.
